# Effects of Fluoro Substitution on the Electrochromic Performance of Alternating Benzotriazole and Benzothiadiazole-Based Donor–Acceptor Type Copolymers

**DOI:** 10.3390/polym10010023

**Published:** 2017-12-25

**Authors:** Yan Zhang, Lingqian Kong, Xiuping Ju, Jinsheng Zhao

**Affiliations:** 1 Shandong Key Laboratory of Chemical Energy Storage and Novel Cell Technology, Liaocheng University, Liaocheng 252059, China; 2 Dongchang College, Liaocheng University, Liaocheng 252059, China; lingqiankong@126.com (L.K.); jxp1127@163.com (X.J.)

**Keywords:** electrochromism, donor–acceptor type polymers, benzotriazole, benzothiadiazole, fluorination

## Abstract

Two new donor–acceptor type electrochromic copolymers containing non-fluorinated and di-fluorinated benzothiadiazole analogues, namely P(TBT-TBTh) and P(TBT-F-TBTh), were synthesized successfully through chemical polymerization. Both polymers were measured by cyclic voltammetry, UV-vis spectroscopy, colorimetry and thermogravimetric analysis to study the influence of fluoro substitution on the electrochromic performance. The results demonstrated that the two polymer films displayed well-defined redox peaks in pairs during the p-type doping, and showed distinct color change from dark gray blue to light green for P(TBT-TBTh) with the band gap of 1.51 eV, and from gray blue to celandine green for P(TBT-F-TBTh) with the band gap of 1.58 eV. P(TBT-F-TBTh) presented lower highest occupied molecular orbital (HOMO) and lowest unoccupied molecular orbital (LUMO) energy levels, and better stability than P(TBT-TBTh). It was found that the two fluorine atoms participated in not only inductive effects but also mesomeric effects in the P(TBT-F-TBTh) backbone. In addition, the polymers exhibited high optical contrasts, short response time, and favorable coloration efficiency, especially in the near infrared region. The characterization results indicated that the two reported polymers can be the potential choice as electrochromic materials.

## 1. Introduction

Conducting polymer is considered as a kind of promising electrochromic material due to its distinctive advantages such as variable color change, high coloration efficiency, excellent switching ability, outstanding cyclic reversibility and low cost [1,2], displaying rapid advances in the technologies of “smart window” [3], electrochromic display device [4], military camouflage [5], electronic paper [6], and so on. The principle of electrochromism is the doping/de-doping process with migration and removal of electrons in the polymer chain which could lead to the transition from valence band to conduction band and cause color changes [7,8]. 

The color contrast depends on the polymer band gap which could be tuned by some effective strategies such as enhancing planarity of the polymer structure, adjusting length of the bonds, changing resonance energy of the polymer backbone, and employing the donor–acceptor (D–A) approach [9,10]. D–A type polymers, with alternating electron-donating and electron-withdrawing units in the backbone, have the inter- or intra-molecular charge transfer (CT) bands as a consequence of push/pull interactions between donor and acceptor. When the interactions are sufficiently intense, the difference of energy levels between highest occupied molecular orbital (HOMO) and lowest unoccupied molecular orbital (LUMO) are reduced accordingly, which can lead to lower band gap and favorable electrochromic properties of the polymers [11,12]. For the sake of further fine-tuning solubility, stability, optical contrast, switching time and coloration efficiency of the electrochromic polymers, as well as color change of RGB (red, green, blue), various strategies of molecule structure design of D–A type polymer have been researched widely [13,14]. Recently, a new kind of donor–acceptor(1)–donor–acceptor(2) (D–A_1_–D–A_2_) type polymer, containing two different alternately arranged acceptors in the polymer backbone, has attracted much attention. This polymer can realize the property complementation of A_1_ and A_2_, and has been developed in the investigation of semiconductor materials to adjust energy levels and extend the absorption region [15,16,17].

Benzotriazole (BTz) is a favorable candidate as acceptor (–C=N as the electron withdrawing group) for D–A type electrochromic polymers owing to the excellent electron transporting ability, ease of synthesis and diverse modification of the structure [18]. Recently, a kind of BTz bearing polymers named poly((2-dodecyl-4,7-di(thiophen-2-yl)-2*H*-benzo[*d*][1,2,3]triazole) (PTBT) took on a significant breakthrough in numerous electrochromic polymers since it displays all red, green, blue (RGB) colors in addition to black and transmissive states [19]. Benzothiadiazole (BTd) has the similar molecular structure but stronger electron accepting ability compared with BTz because of the large polarizability of sulfur atom, which is likely to strengthen the charge transfer process between D and A units and decrease the band gap value of the corresponding polymer [18,20]. In this study, we incorporated BTd units into the PTBT backbone as the second acceptor and synthesized the D–A_1_–D–A_2_ type polymers, expecting to lower band gap and obtain multiple color changes. L. Toppare group synthesized a series of polymers with two acceptors of BTz and benzoquinoxaline, or BTz and benzoselenadiazole, which revealed low optical band gap as 1.48 eV and multiple achievable color changes even black to transmissive switching [21]. In addition, they investigated the copolymer containing alternating BTz and BTd groups and found it showed lower band gap compared to PTBT containing only BTz groups [22]. The other copolymers containing both BTz and BTd groups applied in solar cell and transistors fields have also been researched and reported in the literature [23,24].

Fluorine (F), as a small atom in size with highest Pauling electronegativity of 4.0, can be incorporated onto the electron-withdrawing group of the polymer without deleterious steric effects, and it has been demonstrated a successful strategy to enhance the properties of the polymers [25]. Up to now the researches of conducting polymers containing F atom have ranged over the fields of organic light emitting diodes (OLEDs) [26], polymer solar cell [27], organic field-effect transistors (OFETs) [28], and electrochromic polymers [29]. Owing to the high electronegativity of F atom, the HOMO and LUMO energy levels of the polymers decrease significantly, which would improve the ambient stability and the color persistence in the application [30]. For example, when two F atoms are introduced to the BTz group, the HOMO energy level will be lowered about 0.11 eV predicted by density functional theory (DFT) calculations [31]. Furthermore, the existing interactions between F atoms and neighboring groups such as thiophene rings can enhance the molecular planarity, leading to the closer π–π stacking of polymer chain, which is beneficial to electron transfer during the redox process [30,32].

Herein, encouraged by the above-mentioned researches, we synthesized two D–A_1_–D–A_2_ type electrochromic copolymers namely P(TBT-TBTh) and P(TBT-F-TBTh) with alternate BTz and BTd units as the acceptors, bithiophene units as the donors by Stille cross coupling reaction. BTd group was non-fluorinated in P(TBT-TBTh) structure, while it was di-fluorinated in P(TBT-F-TBTh) structure. The incorporation of long-chain alkyl on BTz and thiophene moieties aimed to enhance the solubility of the polymers. The properties containing electrochemistry, optics, and spectroelectrochemistry affected by the non-fluorinated and di-fluorinated substituent on BTd units were fully investigated.

## 2. Materials and Methods

### 2.1. Materials

1,2,3-Benzotriazole (99%), 1-bromododecane (98%), bis-(triphenylphosphine) dichloropalladium (Pd(PPh_3_)_2_Cl_2_), hydrobromic acid (HBr, 48%), bromine (Br_2_, 99.9%), N-bromosuccinimide (NBS, 99%), tetrabutylammonium hexafluorophosphate (TBAPF_6_, 98%), anhydrous methanol, anhydrous toluene and acetonitrile (ACN) were purchased from Aladdin Chemical Co., Ltd. (Shanghai, China). Chloroform, n-hexane, dichloromethane (DCM), glacial acetic acid (HAC), acetone, magnesium sulfate (MgSO_4_) and sodium hydrogen carbonate (NaHCO_3_) were from Sinopharm Chemical Reagent Co., Ltd. (Shanghai, China). Potassium tert-butoxide (98%) and tributyl(thiophen-2-yl)stannane were purchased from Aldrich Chemical Co., Inc. (Milwaukee, WI, USA). 4,7-bis(4-hexyl-5-(trimethylstannyl)thiophen-2-yl)benzo[*c*][1,2,5]thiadiazole (>98%) and 5,6-difluoro-4,7-bis(4-hexyl-5-(trimethylstannyl)thiophen-2-yl)benzo[*c*][1,2,5]thiadia-zole (>98%) were purchased from Derthon Optoelectronic Materials Science Technology Co., Ltd. (Shenzhen, China). Indium-tin-oxide (ITO) coated glass (sheet resistance: < 10 Ω/sq.), was purchased from Shenzhen CSG Display Technology Co., Ltd. (Shenzhen, China).

### 2.2. Polymer Syntheses

**Scheme 1:**
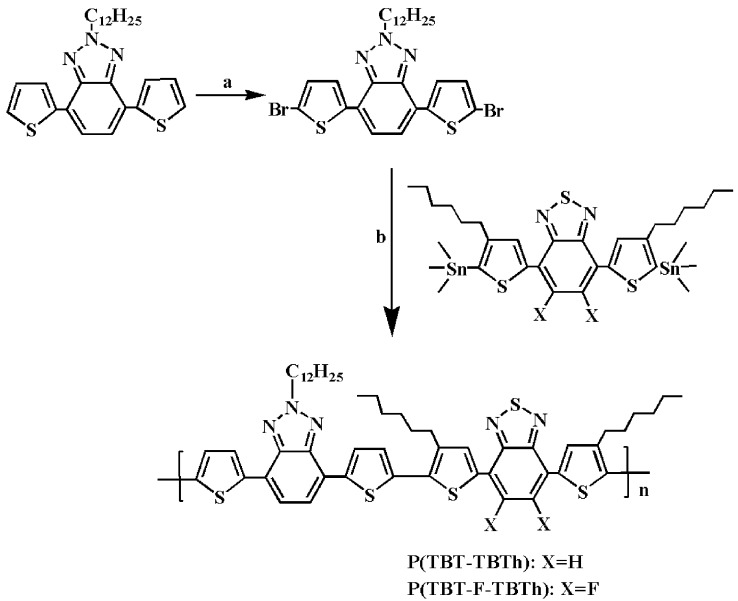
Graphical representation of reaction process of the polymers: (**a**) N-bromosuccinimide (NBS), dichloromethane (DCM)/glacial acetic acid (HAC), refluxed, 12 h; (**b**) Pd(PPh_3_)_2_Cl_2_, toluene, refluxed, 72 h.

The precursor of 2-dodecyl-4,7-di(thiophen-2-yl)-2*H*-benzo[*d*][1,2,3]triazol (TBT) was synthetized according to the literature [33]. The polymer synthetic route was shown in Scheme 1. TBT (1.2 g, 2.66 mmol) and NBS (1.12 g, 6.3 mmol) were added in 60 mL mixed solvent of DCM and HAC (1/1, by volume). The mixture was refluxed gently with stirring for 12 h in the dark environment, and then extracted by DCM and water. After drying and vacuum distilling, the organic phase was refined by column chromatography with hexane/DCM (1:1, by volume) to obtain 1.0 g 2-dodecyl-4,7-di(5-bromo-thiophen-2-yl)-2*H*-benzo[*d*][1,2,3]triazol (fluorescent yellow solid) with 62% yield. ^1^H NMR (400 MHz, CDCl_3_, δ/ppm): 7.78 (d, 2H), 7.50 (s, 2H), 7.12 (d, 2H), 4.78 (t, 2H), 2.18 (t, 2H), 1.56–1.24 (m, 18H), 0.88 (t, 3H). ^13^C NMR (100 MHz, CDCl_3_, δ/ppm): 141.69, 141.20, 130.87, 126.93, 122.97, 122.20, 113.15, 56.94, 31.91, 30.06, 29.62, 29.55, 29.44, 29.35, 29.01, 26.56, 22.70, 14.14 (see Figure S1 in supplementary Materials).

Then, 2-Dodecyl-4,7-di(5-bromo-thiophen-2-yl)-2*H*-benzo[*d*][1,2,3]triazol (0.3 g, 0.5 mmol), 4,7-bis(4-hexyl-5-(trimethylstannyl)thiophen-2-yl)benzo[*c*][1,2,5]thiadiazole (0.4 g, 0.5 mmol) or 5,6-difluoro-4,7-bis(4-hexyl-5-(trimethylstannyl)thiophen-2-yl)benzo[*c*][1,2,5]thiadiazole (0.415 g, 0.5 mmol), and Pd(PPh_3_)_2_Cl_2_ (0.02 g, 0.028 mmol) were dissolved in 100 mL anhydrous toluene. The mixture was heated up to boiling and refluxed 72 h under argon atmosphere. After removing the solvent by vacuum distillation, the crude products were purged through soxhlet extraction by methanol and acetone respectively. By drying and cooling, the purple black products of P(TBT-TBTh) and P(TBT-F-TBTh) were obtained respectively. 

P(TBT-TBTh). ^1^H NMR (600 MHz, CDCl_3_, δ/ppm): 8.18–7.50 (m, 4H), 7.50–6.82 (m, 6H), 5.38–4.79 (m, 2H), 3.73–2.80 (m, 4H), 2.80–1.98 (m, 6H), 1.88–1.07 (m, 30H), 0.98–0.77 (m, 9H) (see Figure S2a in supplementary Materials). The GPC data: M_w_ = 17.0 kDa, M_n_ = 8.8 kDa, polydispersity index = 1.93. 

P(TBT-F-TBTh). ^1^H NMR (600 MHz, CDCl_3_, δ/ppm): 8.28–7.92 (m, 2H), 7.72–7.07 (m, 6H), 4.90–4.79 (m, 2H), 3.67–2.85 (m, 4H), 2.80–1.97 (m, 6H), 1.86–1.06 (m, 30H), 1.01–0.75 (m, 9H) (see Figure S2b in supplementary Materials). The GPC data: M_w_ = 16.7 kDa, M_n_ = 8.9 kDa, polydispersity index = 1.88.

### 2.3. Characterization 

^1^H NMR and ^13^C NMR of the monomer were characterized using a Varian AMX 400 MHz spectrometer (Varian Inc., Santa Clara, CA, USA) with CDCl_3_ as solvent. ^1^H NMR of the polymer was characterized by an Agilent NMR vnmrs 600 MHz spectrometer (Agilent Inc., Palo Alto, CA, USA). Average molecular weights of the two polymers were detected by an alliance GPC 1515 gel permeation chromatography (GPC, Waters Inc., Milford, MA, USA) using tetrahydrofuran as eluent and polystyrene standard as calibrant. Fourier transform infrared (FT-IR) spectra were measured on a Nicolet 6700 FTIR spectrometer (Thermo Fisher Scientific Inc., Waltham, MA, USA). 

The electrochemical experiments were executed in a self-made electrochemical cell serviced by a CHI 760 C electrochemistry workstation (Shanghai Chenhua Instrument Co., Shanghai, China). Before experiments, the polymer solution was prepared with the concentration of 5 mg/mL (solvent: chloroform), and then sprayed on the ITO glass (the active area: 0.9 cm × 3.0 cm) by the airbrush to form a membrane when the solvent evaporated thoroughly. The ITO glass coated with polymer was used as working electrode (WE); meanwhile, a platinum ring was used as counter electrode (CE), and a silver wire (0.03 V vs. saturated calomel electrode, SCE) as pseudo-reference electrode (RE). 

Spectroelectrochemical experiments were carried out using a Varian Cary 5000 spectrophotometer (Varian Inc., Santa Clara, CA, USA). The potentials applied on the polymer film was controlled by the electrochemical experiments mentioned above employing a ITO glass (coated with polymer), a stainless-steel wire and a silver wire as WE, CE and RE, respectively. Colorimetry was also surveyed by the Varian Cary 5000 spectrophotometer.

Microscopic morphology was determined by a ZEISS scanning electron microscope (SEM, Carl Zeiss Ltd., Oberkochen, Germany). The thicknesses of the polymer films were measured by a KLA-Tencor D-100 step profiler (KLA-Tencor Inc., Milpitas, CA, USA). Digital images of the polymer films were taken by a 24 million pixels digital camera (Canon Inc., Tokyo, Japan).

Thermogravimetric analysis (TGA) was tested by a Netzsch STA449C TG/DSC simultaneous thermal analyzer (Netzsch Inc., Bavaria, Germany) using approximately 5 mg samples which were heated from 0 to 800 °C in nitrogen atmosphere with 10 °C/min heating rate.

## 3. Results and discussion

### 3.1. FT-IR Spectra 

**Figure 1:**
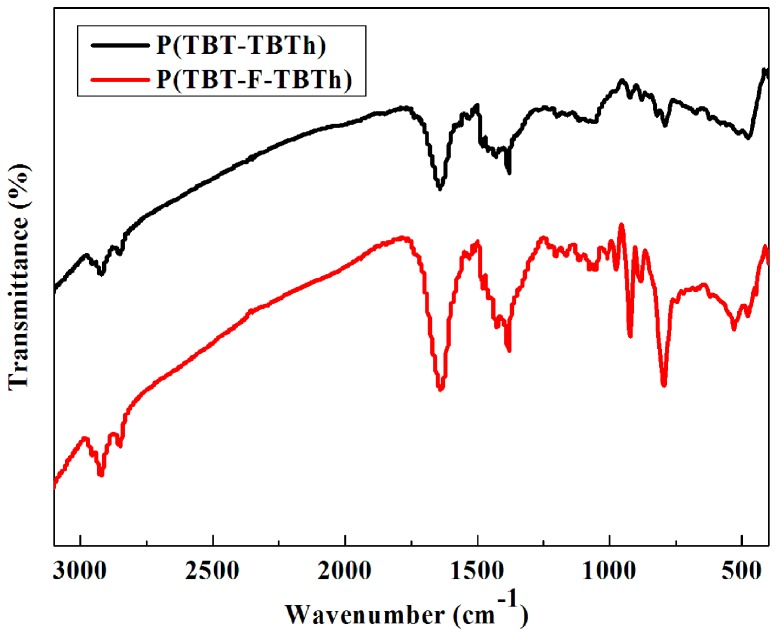
The FT-IR spectra of P(TBT-TBTh) and P(TBT-F-TBTh).

The FT-IR spectra of P(TBT-TBTh) and P(TBT-F-TBTh) were shown in Figure 1. The following peaks were identified in the spectrum of P(TBT-TBTh): 2922 cm^–1^ (aliphatic C–H stretching vibration), 1637 cm^–1^ (phenyl substituted ring C=C stretching vibration), 1492–1390 cm^–1^ (thiophene substituted ring C=C and triazole C–N stretching vibration), 1384 cm^–1^ (methyl C–H symmetrical bending vibration), 1060 cm^–1^ (triazole N–N stretching vibration), 790 cm^–1^ (phenyl and thiophene substituted ring C–H out of plane bending vibrations). The aforementioned peaks could also be observed in the FT-IR spectrum of P(TBT-F-TBTh). In addition, compared with P(TBT-TBTh), P(TBT-F-TBTh) displayed a new band centered at 923 cm^–1^ which was ascribed to stretching vibration of C–F bond in phenyl substituted ring.

### 3.2. Electrochemistry 

**Figure 2:**
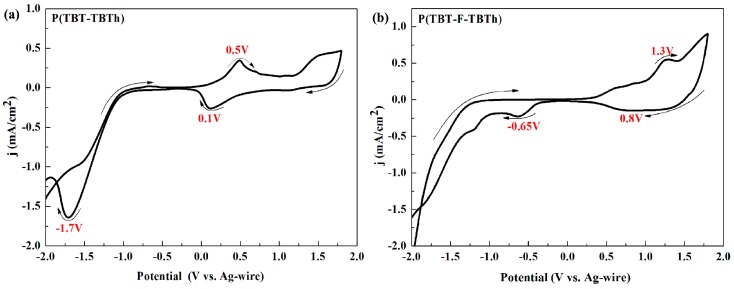
Cyclic voltammetry (CV) curves of the polymer films: (**a**) P(TBT-TBTh); (**b**) P(TBT-F-TBTh).

Electrochemical characterization was detected through cyclic voltammetry (CV) experiments. After the solution of P(TBT-TBTh) and P(TBT-F-TBTh) were spray-coated onto the ITO glasses and obtaining the polymer thin films, CV tests were performed in ACN solution containing 0.1 M TBAPF_6_ as the electrolyte at 100 mV/s scan rate with the potentials between 2.0 and −2.0 V. As shown in Figure 2, the two polymers had obvious redox peaks in pairs at the positive potential, i.e., better p-type doping occurred. The oxidation and reduction potentials were shown by the peaks at 0.50 and 0.10 V for P(TBT-TBTh), at 1.30 and 0.8 V for P(TBT-F-TBTh), respectively. The onset oxidation potentials (*E*_onset_) of P(TBT-TBTh) and P(TBT-F-TBTh) were respectively 0.19 and 0.84 V. However, from the CV diagram of negative potential, their n-type doping was not very obvious with only a clear reduction peak at −1.70 V for P(TBT-TBTh) and −0.65 V for P(TBT-F-TBTh) due to the weak stability and storage capacity of the radical anion produced from n-type doping process. According to reports in the literatures, highly fluorinated polymers like tetradecafluorosexithiophene [34] and perfluoropentacene [35], as well as perfluoroalkyl end-capped oligothiophenes [36] could exhibit excellent n-type doping as semiconductors, while fluoroarene intermediate-embedded oligothiophenes with fewer F atoms [37] show inconspicuous or no n-type doping. The experimental results of P(TBT-TBTh) and P(TBT-F-TBTh) with intense p-type doping and weak n-type doping coincided well with the above references.

By contrast, it was easy to find that the redox potentials and *E*_onset_ of P(TBT-F-TBTh) were higher than that of P(TBT-TBTh), which was mainly attributed to the existence of F groups with high electronegativity. When F groups were linked to the BTd conjugate unit, there was an inductive effect which would hinder the transfer of donor electrons in the p-type doping process, resulting in an increase of the potentials [29,38]. In addition, during the negative scan, the reduction potential of P(TBT-F-TBTh) was also higher than that of P(TBT-TBTh) since the strong electron-withdrawing F groups of P(TBT-F-TBTh) made the electrons were more easily transferred from the electrode to the polymer in the n-type doping process.

### 3.3. Morphology

**Figure 3:**
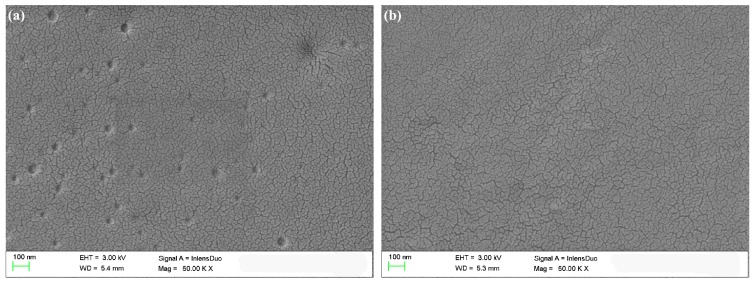
SEM images of the polymer films with magnification of 50000: (**a**) P(TBT-TBTh); (**b**) P(TBT-F-TBTh).

**Figure 4:**
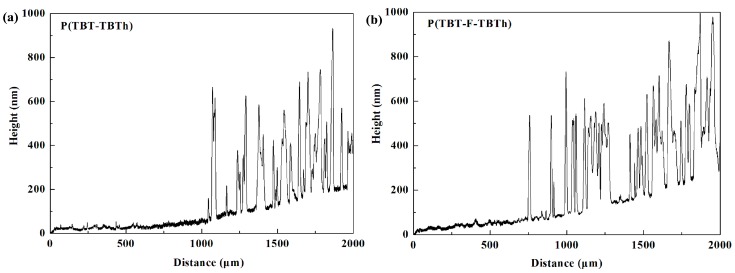
Film thicknesses of the polymers spray-coated onto the indium-tin-oxide (ITO) glasses: (**a**) P(TBT-TBTh); (**b**) P(TBT-F-TBTh).

After the polymers were sprayed on the ITO glasses, SEM measurements were performed to observe the microstructure and bulk morphologies of the films. As shown in Figure 3, the SEM images of P(TBT-TBTh) and P(TBT-F-TBTh) at 50,000 magnification were similar. Separated particles of the polymers were present as nano-scale flakes which were distributed uniformly on the film surfaces with irregular shapes, and a large number of curved stripes were displayed. In addition, the thicknesses of the polymer films were tested as illustrated in Figure 4. It can be seen that multiple peaks were arranged irregularly in the spectra, and each peak represented the polymer particles coated on the ITO glasses. The average height of the peaks namely the thickness of the coated polymer particles was measured as about 560 nm for P(TBT-TBTh) and 570 nm for P(TBT-F-TBTh). The thickness of the whole film including particles and stripes was calculated as 232 nm for P(TBT-TBTh) and 257 nm for P(TBT-F-TBTh). The rough curves of the thicknesses implied the irregular and discontinuous surfaces of the two polymer films, which was in accord with the morphologies given by the SEM experiments.

### 3.4. Optical Properties 

**Figure 5:**
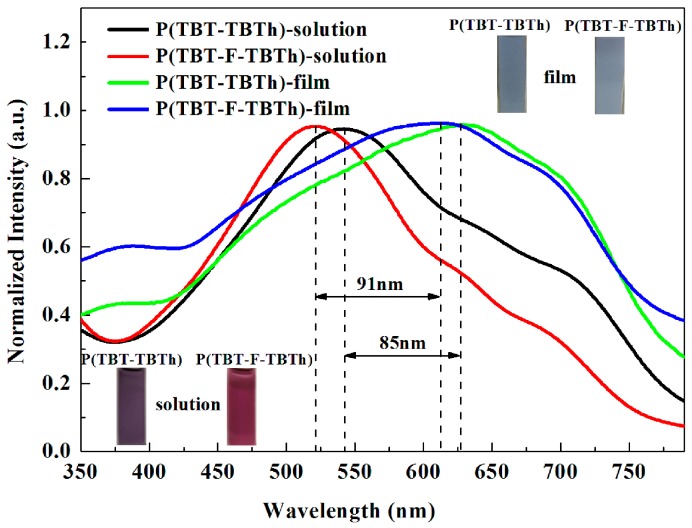
UV-vis spectra of the polymers in chloroform saturated solution and in thin film.

The UV-vis absorption spectra of the two polymers were studied in chloroform saturated solution and in solid film (spray-coated on the ITO glasses), respectively. Figure 5 showed the optical properties and the corresponding colors of the polymers. Each polymer displayed a unique absorption band which was attributed to the π–π* transition [39]. The absorption peak of P(TBT-TBTh) film at 627 nm showed much broader and 85 nm bathochromic-shift compared with that of P(TBT-TBTh) solution at 542 nm. The corresponding color was also different: the saturated solution was purple, and the neutral film became dark grey blue. Similarly, the peak of P(TBT-F-TBTh) displayed evidently broadened absorption and 91 nm bathochromic-shift in the solid state (612 nm) with respect to in the solution (521 nm). Also, the color of the saturated solution was wine red, while the neutral film was grey blue. The reason for the bathochromic-shift phenomena was the strong intermolecular interaction caused by the effective planarization and the closer packing of the polymer backbones in the thin film [25,38,40]. Both in the solution and in the solid film, the absorption peaks of P(TBT-F-TBTh) exhibit hypsochromic shift compared to that of P(TBT-TBTh), which could be explained by the conjugative effect and the inductive effect. In fact, besides the π–π conjugation of polymer chain, the introduction of two F atoms would induce p–π conjugative effect, also this effect was very weak. In addition, F atoms could enhance the planarity of P(TBT-F-TBTh) backbone via the interactions of C–F···H, F···S, and C–F···π_F_, which would further improve the conjugative effect to a certain extent [32]. However, due to the high electronegativity and strong electron-withdrawing ability of F atoms, the inductive effect would be greater than the p–π conjugative effect. Hence, the electronic cloud density of conjugated chain declined, resulting in the reduced conjugation length and the hypsochromic shift phenomena.

### 3.5. Spectroelectrochemistry

**Figure 6:**
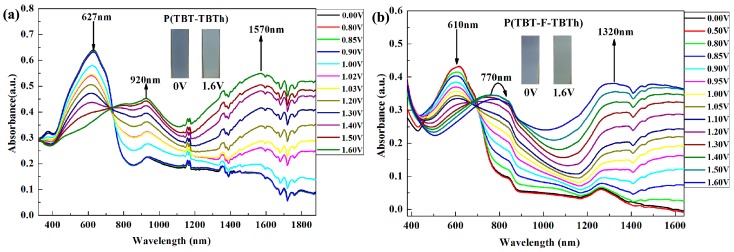
Spectroelectrochemical spectra of the polymer films: (**a**) P(TBT-TBTh); (**b**) P(TBT-F-TBTh).

In order to explore the evolution of the charge carries in the polymer chains, spectroelectrochemical studies of the polymer film (spray-coated onto the ITO glasses beforehand) were carried out at the desired potential ranging from 0 to 1.6 V in a 0.1 M TBAPF_6_/ACN solution. The full-detailed absorption spectra and the corresponding colors of P(TBT-TBTh) and P(TBT-F-TBTh) were shown in Figure 6. 

At the neutral state, the films displayed absorption peak at 627 nm for P(TBT-TBTh) and 610 nm for P(TBT-F-TBTh) in the visible region arising from π–π* transition in the polymer backbone. With the increase of potential, the absorption bands of the neutral state graduated away, and the other two new absorption bands in the near-infrared region (NIR) heightened simultaneously, implying the formation of the lower energy charge carriers ascribed to the evolution of polaron and bipolaron bands [39]. For P(TBT-TBTh), as shown in Figure 6a, π–π* transition band disappeared thoroughly when fully oxidized to 1.6V. In the meantime, the new absorption peaks at around 920 nm and 1570 nm, corresponding to the polaron and bipolaron bands respectively, gradually increased monotonically until reached to the maximum. Nevertheless, the π–π* transition bands of P(TBT-F-TBTh) attenuated entirely at 1.4 V as Figure 6b suggested, meanwhile, the new peak of polaron band at 770 nm increased to maximum. When fully oxidized to 1.6 V, the other low-energy peak at around 1320 nm ascribed to the bipolaron band achieved its maximum. In particular, the polaron band weakened gradually from 1.4 to 1.6 V because the continuously enhancing bipolaron band restrained the polaronic population which was also described as the repression of interband transition [41]. Some other polymers also possess the properties similar to P(TBT-F-TBTh), that is they would lead to spin-1/2 polarons at light doping and blossom into spinless bipolarons at heavy doping [42]. In addition, it was noteworthy that both the π–π* transition band and the polaron/bipolaron band of P(TBT-F-TBTh) showed evident hypsochromic shift compared with those of P(TBT-TBTh) due to the verified inductive effect which proceeded from the F groups in P(TBT-F-TBTh) backbone. With the attenuation of the high-energy absorption bands at neutral state and the enhancement of the two low-energy absorption bands at oxidized state, P(TBT-TBTh) showed color change from dark gray blue to light green, and P(TBT-F-TBTh) displayed from gray blue to celandine green.

Some of the spectroscopic characterization parameters of P(TBT-TBTh) and P(TBT-F-TBTh) including maximum absorption wavelength (λ_max_), low energy onset absorption wavelength at the neutral state (λ_onset_), *E*_onset_, optical band gap (*E*_g,op_), HOMO and LUMO energy levels, which were gained directly from the graph or calculated by a certain method, were sorted into Table 1. It can be seen that the *E*_g,op_ of P(TBT-TBTh) and P(TBT-F-TBTh), producing from the different λ_onset_ values, were 1.51 and 1.58 eV respectively, which were both lower than that of PTBT (only including BTz groups, 1.65 eV) due to the BTd groups existing in P(TBT-TBTh) and P(TBT-F-TBTh) with stronger electron accepting ability [19]. In the other aspect, the presence of two electron withdrawing F groups in BTd unit made it difficult to generate oxidizing reaction in the doping process, leading to the higher *E*_g,op_ of P(TBT-F-TBTh). The HOMO energy levels of P(TBT-TBTh) and P(TBT-F-TBTh) were calculated as −4.61 and −5.26 eV respectively according on their *E*_onset_, which was agree with the previous report that fluorination can effectively reduce the HOMO energy level of polymer and in turn strengthen the stability by lowering the propensity towards oxidative degradation [27,29,30,31,38,40]. Similarly, P(TBT-F-TBTh) also exhibited lower LUMO energy level than P(TBT-TBTh).

To further estimate the optoelectronic properties of the two polymers, the DFT calculations were carried out by Gaussian 09 software to obtain the theoretical value of HOMO and LUMO energy levels which were listed in Table 1. The simulated data of HOMO and LUMO energy levels were −5.10 and −3.17 eV for P(TBT-TBTh), and −5.19 and −3.39 eV for P(TBT-F-TBTh), which had a certain error compared with the experimental data. However, what accorded with the experimental results was that the fluorinated polymer also revealed lower HOMO and LUMO energy levels than nonfluorinated polymer. 

**Figure 7:**
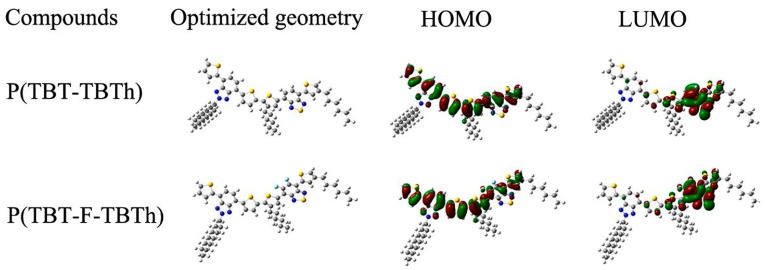
Optimized geometries and molecular orbital surfaces of the HOMO and LUMO for P(TBT-TBTh) and P(TBT-F-TBTh).

The configuration and optimization of electronic cloud distribution of the two polymers were depicted in Figure 7. It was observed that a delocalized HOMO was equally distributed along the whole conjugated backbone; the LUMO, however, was mainly localized on the BTd acceptor groups, which proved the well-defined D–A type conjugated structure and the effective intramolecular charge transfer from donor to acceptor [27,40]. The LUMO was found concentrated on the BTd part rather than on the BTz part, which can be explicated by the stronger electron accepting ability of BTd than BTz. As shown in the figure of P(TBT-F-TBTh), F atoms were distributed on both HOMO and LUMO, which was inconsistent with the D–A nature property that the HOMO was related with the donor unit and the LUMO with the acceptor unit. In other words, F atoms should be covered mainly on the LUMO attributed to its electron withdrawing effect. Thus, the actual distribution suggested that, besides the inductive withdrawing properties, F atom also participated in the mesomeric donation effects from its lone pairs [29,38]. Finally, the mesomeric effects were weaker than the inductive effects because the contribution of F atoms to the HOMO was smaller than that to the LUMO.

### 3.6. Electrochromic Switching 

**Figure 8:**
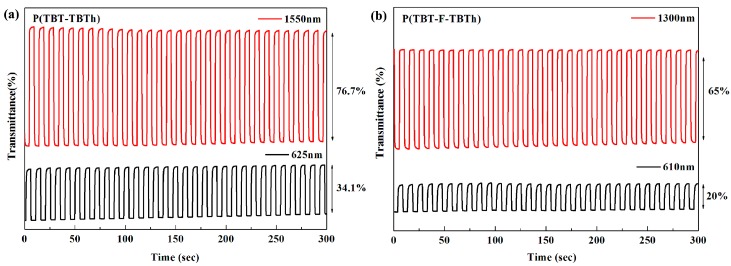
Electrochromic switching of the polymer films: (**a**) P(TBT-TBTh); (**b**) P(TBT-F-TBTh).

It is very important for electrochromic polymers to have fast switching property and rapid distinct color change when the polymer films change between neutral state and oxidized state. Dynamic electrochromic experiments were performed in ACN solution containing 0.1 M TBAPF_6_ electrolyte under the square wave potential with regular interval of 5 s between 0 V and 1.6 V using the spectrophotometer coupled with the double potential step chronoamperometry of electrochemical workstation. The polymers were beforehand spray-coated onto the ITO glasses. The detection wavelengths were chosen the absorption maximum (λ_max_) on the basis of spectroelectrochemical spectra, settled at 625 and 1550 nm for P(TBT-TBTh), and at 610 nm and 1300 nm for P(TBT-F-TBTh), respectively. Figure 8 demonstrated the relations of optical contrast versus time. Subsequently the optical contrast, the response time and the coloration efficiency were all calculated and listed in Table 2. 

Optical contrast (*T*%), may be defined as transmittance change between the neutral state and the oxidized state, was determined as 34.1% at 625 nm, 76.7% at 1550 nm for P(TBT-TBTh), and as 20% at 610 nm, 65% at 1300 nm for P(TBT-F-TBTh), respectively. 

Response time (*t*_95%_), reflecting the switching speed between the redox states, is regarded as the time reaching 95% of the full optical switch. The *t*_95%_ of P(TBT-TBTh) were calculated to be 0.85 s at 625 nm and 0.70 s at 1550 nm from neutral state to oxidation state, and to be 0.32 s at 625 nm and 0.60 s at 1550 nm from oxidation state to neutral state. While for P(TBT-F-TBTh), the *t*_95%_ were determined as 1.75 s at 610 nm and 0.71 s at 1300 nm from neutral state to oxidation state, and as 0.73 s at 610 nm and 0.40 s at 1300 nm from oxidation state to neutral state. For the two polymers, it was found that the oxidation process was slower than the reduction process since the counter ions of TBAPF_6_ electrolyte presented different diffusion rates at different charged states of the polymer films [20].

Coloration efficiency (*CE*) is the parameter about power efficiency for the electrochromic process, which can be calculated by the equations [43]:

$$\Delta OD=\log(\frac{T_{b}}{T_{c}});\;CE=\frac{\Delta OD}{\Delta Q}$$

where Δ*OD* is optical density change, *T*_b_ and *T*_c_ are *T*% before and after coloration respectively, and Δ*Q* is charge consumed per unit electrode area. Through calculation using the above formulas, the *CE* of P(TBT-TBTh) were 128.9 cm^2^/C at 625 nm and 258.5 cm^2^/C at 1550 nm, and that of P(TBT-F-TBTh) were 90.8 cm^2^/C at 610 nm and 197.8 cm^2^/C at 1300 nm. 

By comparing the kinetic parameters in the visible region and in the NIR region, it can be seen that the two polymers had greater optical contrast, shorter response time and higher coloration efficiency in the NIR region, suggesting potential candidates in the utilization of NIR electrochromic materials. On the other hand, in contrast to P(TBT-F-TBTh), P(TBT-TBTh) displayed better properties in respect of *T*%, *t*_95%_ (oxidation process), and *CE*, which was the result of the faster dopant ion diffusion velocity and the easier charge transfer in the P(TBT-TBTh) backbone without inductive effect of F atoms during the doping/de-doping processes.

**Figure 9:**
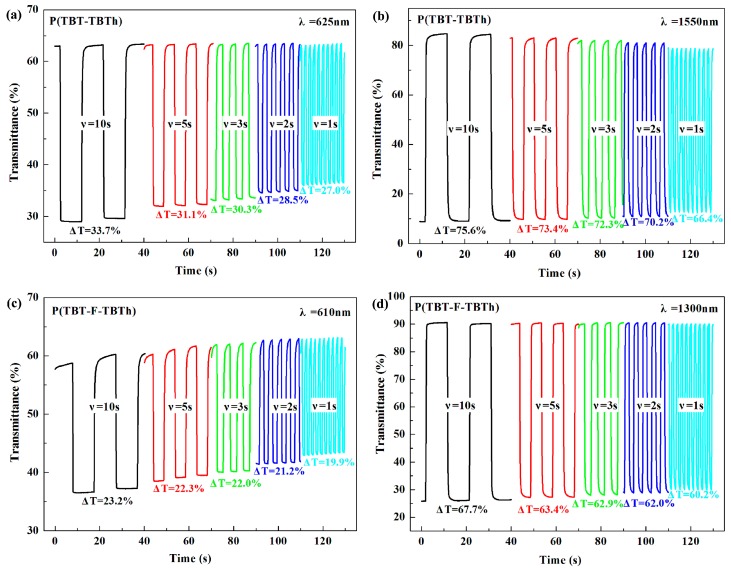
Electrochromic switching of the polymer films with different intervals: (**a**) P(TBT-TBTh) monitored at 625 nm; (**b**) P(TBT-TBTh) monitored at 1550 nm; (**c**) P(TBT-F-TBTh) monitored at 610 nm; (**d**) P(TBT-F-TBTh) monitored at 1300 nm.

Furthermore, another method of dynamics research was performed by changing the time interval of multistep potentials from 10 to 5, 3, 2 s and finally to 1 s. During the test, the other experimental conditions and selected wavelengths were same as the above electrochromic switching experiment. As shown in Figure 9, with the time interval changing from 10 s to 1 s, the *T*% of P(TBT-TBTh) decreased by 6.7% at 625 nm and 9.2% at 1550 nm respectively, and that of P(TBT-F-TBTh) decreased by 3.3% at 610 nm and 7.5% at 1330 nm respectively. The above results revealed that P(TBT-F-TBTh) had better stability and color persistence than P(TBT-TBTh) because the interaction of F atoms with the neighboring atoms could improve the planarity of polymer backbones, leading to the closer π–π polymer chain stacking in the P(TBT-F-TBTh) structure [32]. In addition, the lower HOMO and LUMO energy levels engendered by the electron-withdrawing F groups also indicated better stability of P(TBT-F-TBTh) [32,38,40].

### 3.7. Colorimetry

Through the spectroelectrochemistry experiments, it was found that the color change of P(TBT-TBTh) and P(TBT-F-TBTh) was not significantly different during the redox process observed by the naked eye. For the sake of the detail of color change in the electrochemical conversion of the polymers, colorimetric experiments were conducted to determine the L*, a* and b* values at the suitable potential from 0 to 1.7 V in a 0.1 M TBAPF_6_/ACN solution by combining CIE 1976 L* a* b* Color Space software and Varian Cary 5000 spectrophotometer. Before experiments, the polymers were spray-coated on the ITO glasses. In L* a* b* Color Space, L* denotes the lightness of color from black to white with the value from 0 to 100, a* denotes red–green balance which means the positive value represents red and the negative value represents green, and b* denotes yellow–blue balance which means the positive value represents yellow and the negative value represents blue [44]. 

**Figure 10:**
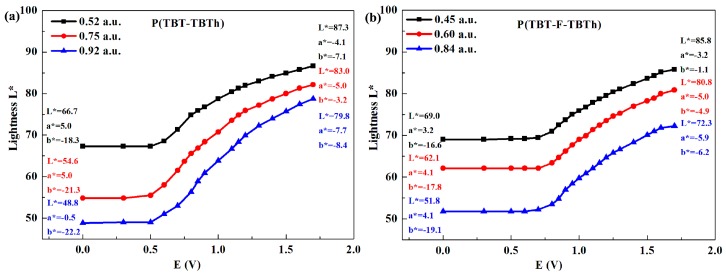
Plot of lightness of the polymer films as a function of applied potential: (**a**) P(TBT-TBTh); (**b**) P(TBT-F-TBTh).

Each polymer film was subjected to three experiments with different thickness which were expressed in the maximum optical absorption values of the polymers, and the functional relationship between L* and applied potential as well as the values of L*, a* and b* at 0 and 1.7 V were illustrated in Figure 10. For the two polymers, it was obviously observed that the L* value firstly remained unchanged as the applied potential increased, and then gradually augmented after the potential exceeded *E*_onset_. Meanwhile, the value of a* and b* were also changed in the oxidation process. For example, the a* value of P(TBT-TBTh) with the maximum absorption of 0.52 a.u. (the thinnest film) changed from a small positive to a negative, while the b* value raised from a large negative to a small negative, which indicated the color turned from blue to green as shown in Figure 6a. The a* and b* values of P(TBT-F-TBTh) displayed analogous changes compared with those of P(TBT-TBTh). 

Moreover, with the increase of film thickness, that is the increase of maximum optical absorption values, the L* value decreased whether at the neutral state or the oxidized state, which indicted the thinner the film was, the higher the brightness was. For P(TBT-TBTh), as the maximum absorption increased from 0.52 to 0.75, and to 0.92 a.u., the L* values were measured as 66.7, 54.6 and 48.8 at the neutral state, and as 87.3, 83.0 and 79.8 at the oxidized state, respectively. In addition, for P(TBT-F-TBTh), as the maximum absorption changed from 0.45 to 0.60, and to 0.84 a.u., the L* values were 69.0, 62.1 and 51.8 at the neutral state, and 85.8, 80.8 and 72.3 at the oxidized state, respectively. Therefore, the brightness can be turned by adjusting the film thickness in practical applications, which had an important reference value for the studies on high-resolution displays and electrochromic smart window.

### 3.8. Thermogravimetric Analysis

**Figure 11:**
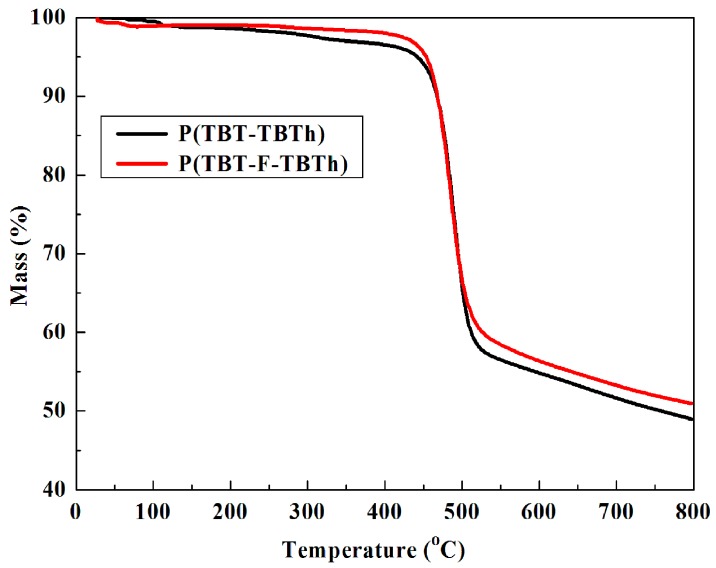
Thermogravimetric analysis (TGA) of P(TBT-TBTh) and P(TBT-F-TBTh).

The thermal stability of electrochromic materials, as an important characteristic in the applications, can be examined by TGA technique [40]. As shown in Figure 11, the weight of P(TBT-TBTh) and P(TBT-F-TBTh) showed no significant loss below 420 °C, suggesting satisfactory thermal stability, which was in turn beneficial to enhance the morphological stability and the service time in device utilization. The decomposition temperature (T_d_) at 5% weight loss of P(TBT-TBTh) and P(TBT-F-TBTh) were recorded at 442.3 and 453.4 °C, respectively. The char yield, representing amount of carbonized residue, were 48.9% for P(TBT-TBTh) and 50.7% for P(TBT-F-TBTh) at 800 °C, and the relatively high values were ascribed to the abundant aromatic groups distributed in the structure. By comparing the above data, it was concluded that P(TBT-F-TBTh) exhibited a more favorable thermal stability than P(TBT-TBTh), which approved the opinion in the literature that the direct fluorine substitution on the polymer backbone could improve the thermal stability of the polymers [32].

## 4. Conclusions 

In summary, two novel electrochromic copolymers, P(TBT-TBTh) and P(TBT-F-TBTh) were prepared through Stille crossing-coupling reaction. F atoms substitution on the BTz group had a significant effect on the properties of polymer. The two polymers showed obvious color transition which was exhibited from dark gray blue to light green by P(TBT-TBTh) and from gray blue to celandine green by P(TBT-F-TBTh) during the redox processes. Compared with P(TBT-TBTh), P(TBT-F-TBTh) had higher band gap but lower HOMO and LUMO energy levels, and displayed better ambient stability, color persistence and thermal stability. Moreover, compared with in the visible region, the two polymers presented better properties in the NIR region such as more desirable optical contrast, shorter response time and more attractive coloration efficiency. By the above positive results, P(TBT-TBTh) and P(TBT-F-TBTh) demonstrated respective unique characteristics and different advantages and deserve further research to obtain more promising electrochromic materials.

## Figures and Tables

**Table 1:** Optical and electrochemical data of P(TBT-TBTh) and P(TBT-F-TBTh).

Polymers	λ_max_ (solutio)	λ_max_ (film)	λ_onset_ (film)	*E*_onset_	*E*_g,op_^a^	HOMO ^b^	LUMO ^c^	HOMO_cal_^d^	LUMO_cal_^d^
	(nm)	(nm)	(nm)	(V)	(eV)	(eV)	(eV)	(eV)	(eV)
P(TBT-TBTh)	542	627	820	0.19	1.51	−4.61	−3.10	−5.10	−3.17
P(TBT-F-TBTh)	521	612	785	0.84	1.58	−5.26	−3.68	−5.19	−3.39

^a^ *E*_g,op_ = 1241/λ_onset_;

^b^ Highest occupied molecular orbital (HOMO) = −e(*E*_onset_ + 0.02 + 4.4) (vs. SCE), 0.02 is to calibrate the error of reference electrode;

^c^ Lowest unoccupied molecular orbital (LUMO) = HOMO + *E*_g,op_;

^d^ HOMO_cal_ and LUMO_cal_ were calculated by DFT calculations.

**Table 2:** Δ*T*%, *t*_95%_ and CE of P(TBT-TBTh) and P(TBT-F-TBTh) at their corresponding wavelengths.

Copolymers	λ (nm)	Δ*T*% (%)	*t*_95%_ (s)		Δ*OD*	*CE* (cm^2^/C)
			Neutral to oxidation	Oxidation to neutral		
P(TBT-TBTh)	625	34.1	0.85	0.32	0.35	128.9
	1550	76.7	0.70	0.60	0.89	258.5
P(TBT-F-TBTh)	610	20.0	1.75	0.73	0.21	90.8
	1300	65.0	0.71	0.40	0.50	197.8

## Supplementary Materials

The following are available online at www.mdpi.com/2073-4360/10/1/23/s1, Figure S1: ^1^H NMR spectrum and ^13^C NMR spectrum of 2-dodecyl-4,7-di(5-bromo-thiophen-2-yl)-2*H*-benzo[*d*][1,2,3]triazol in CDCl_3_: (a) ^1^H NMR, solvent peak at δ = 7.26 ppm was marked by “x”; (b) ^13^C NMR, solvent peak at δ = 77.01 ppm was marked by “X”. Figure S2. ^1^H NMR spectra of the polymers in CDCl_3_: (a) P(TBT-TBTh); (b) P(TBT-F-TBTh). Solvent and tetramethylsilane peaks were marked by “x”, “y” respectively.
