# Supplementary material for: Effects of Fluoro Substitution on the Electrochromic Performance of Alternating Benzotriazole and Benzothiadiazole-Based Donor–Acceptor Type Copolymers
Source: Polymers (Basel). 2017 Dec 25;10(1):23. doi: 10.3390/polym10010023 (PMC6414881; doi:10.3390/polym10010023)
Supplement: Supplementary file 1 [file polymers-10-00023-s001.pdf]

Effects of Fluoro Substitution on the Electrochromic Performance of Alternating Benzotriazole and Benzothiadiazole-Based Donor–Acceptor Type Copolymers

Yan Zhang[1,*], Lingqian Kong[2], Xiuping Ju[2], Jinsheng Zhao[1,*]

[1] Shandong Key Laboratory of Chemical Energy Storage and Novel Cell Technology, Liaocheng University, Liaocheng, 252059, China

[2] Dongchang College, Liaocheng University, Liaocheng, 252059, China; lingqiankong@126.com (L.K.); jxp1127@163.com (X.J.)

* Correspondence: zy@lcu.edu.cn (Y.Z.); j.s.zhao@163.com (J.Z.);

Tel.: +86-635-823-9325 (Y.Z.); Tel.: +86-635-853-9607 (J.Z.)

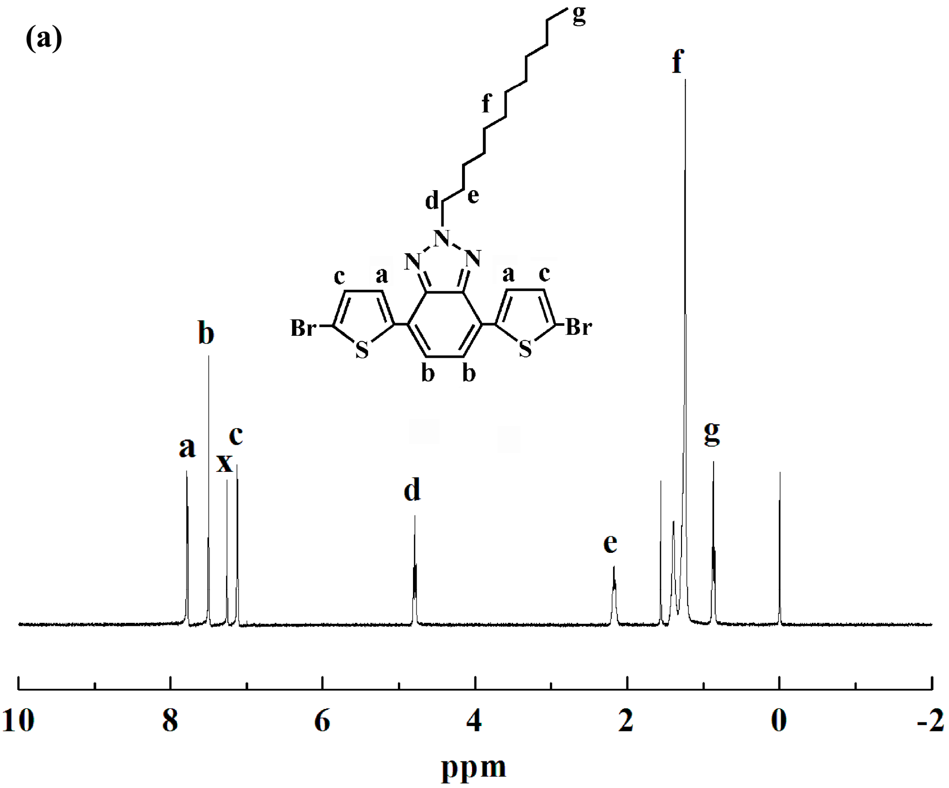

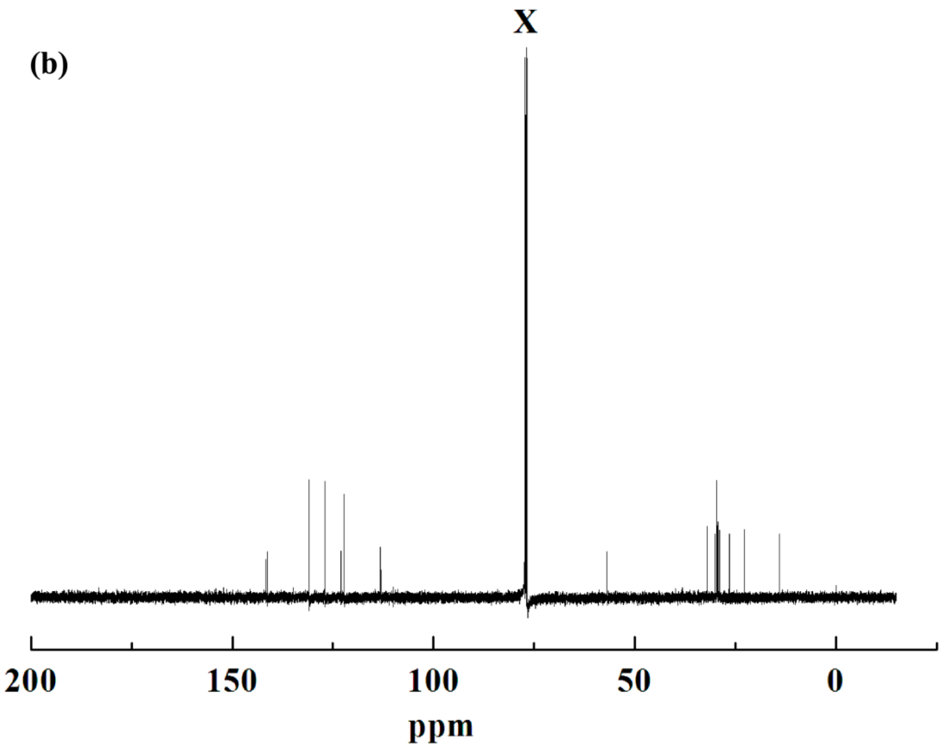

Figure S1. ^{1}H NMR spectrum and ^{13}C NMR spectrum of 2-dodecyl-4,7-di(5-bromo-thiophen-2-yl)-2H-benzo[d][1,2,3]triazol in $CDCl_3$: (a) ^{1}H NMR, solvent peak at $\delta = 7.26$ ppm was marked by "x". (b) ^{13}C NMR, solvent peak at $\delta = 77.01$ ppm was marked by "X".

(a)

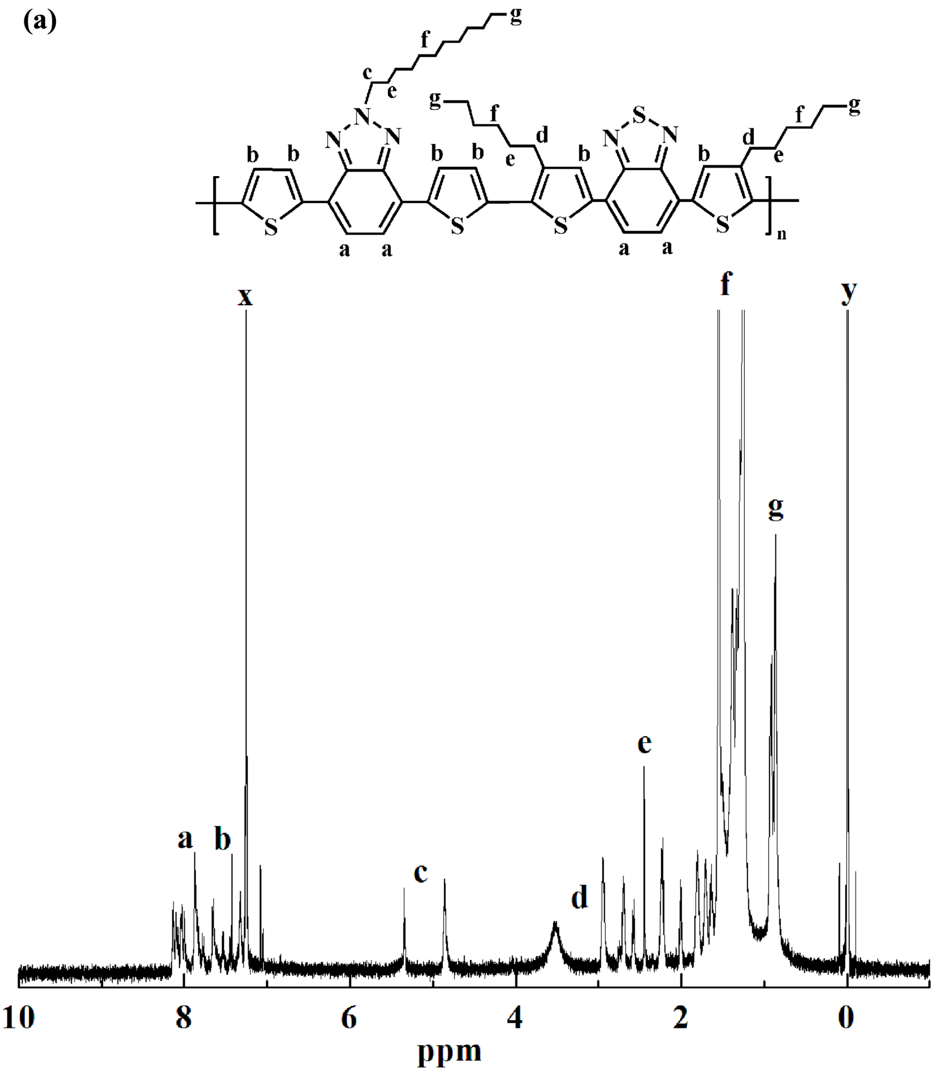

(b)

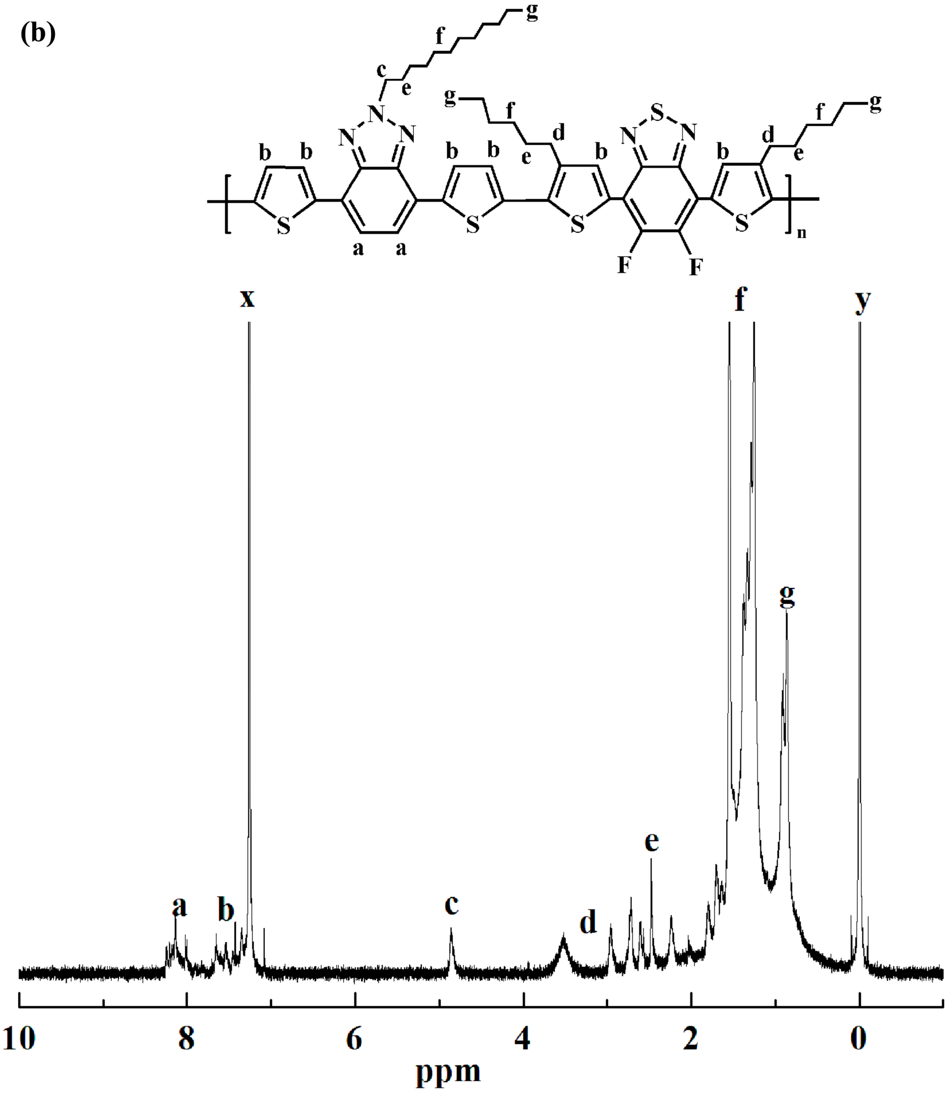

Figure S2. ^{1}H NMR spectra of the polymers in $CDCl_3$: (a) P(TBT-TBTh); (b) P(TBT-F-TBTh). Solvent and tetramethylsilane peaks were marked by “x”, “y” respectively.
